# *r*-2,*c*-6-Bis(4-methoxy­phenyl)-*c*-3,*t*-3-dimethyl-1-nitro­sopiperidin-4-one

**DOI:** 10.1107/S1600536809019357

**Published:** 2009-05-29

**Authors:** T. Kavitha, S. Ponnuswamy, P. Sakthivel, K. Karthik, M. N. Ponnuswamy

**Affiliations:** a Centre of Advanced Study in Crystallography and Biophysics, University of Madras, Guindy Campus, Chennai 600 025, India; b Department of Chemistry, Government Arts College (Autonomous), Coimbatore 641 018, Tamil Nadu, India

## Abstract

In the title compound, C_21_H_24_N_2_O_4_, the piperidine ring adopts a distorted boat conformation. The crystal structure is stabilized by C—H⋯π inter­actions involving one of the methoxy­phenyl rings.

## Related literature

For the biological activity of piperidones, see: Dimmock *et al.* (1990 ▶); Mutus *et al.* (1989 ▶); Perumal *et al.* (2001 ▶). For ring conformations, see: Cremer & Pople (1975 ▶); Nardelli (1983 ▶).
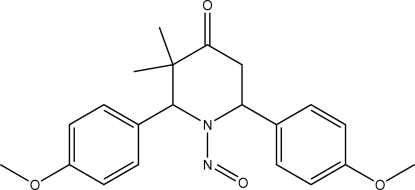


## Experimental

### 

#### Crystal data

C_21_H_24_N_2_O_4_
*M* *_r_* = 368.42
Orthorhombic, 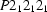

*a* = 7.2540 (3) Å
*b* = 15.0469 (6) Å
*c* = 17.0741 (7) Å
*V* = 1863.64 (13) Å^3^
*Z* = 4
Mo *K*α radiation
μ = 0.09 mm^−1^
*T* = 293 K
0.30 × 0.25 × 0.20 mm

#### Data collection

Bruker Kappa APEXII CCD area-detector diffractometer
Absorption correction: multi-scan (*SADABS*; Sheldrick, 2001 ▶) *T*_min_ = 0.973, *T*_max_ = 0.982
24656 measured reflections
3211 independent reflections
2595 reflections with *I* > 2σ(*I*)
*R*_int_ = 0.026

#### Refinement

*R*[*F* ^2^ > 2σ(*F* ^2^)] = 0.039
*wR*(*F* ^2^) = 0.109
*S* = 1.03
3211 reflections
244 parameters
H-atom parameters constrained
Δρ_max_ = 0.20 e Å^−3^
Δρ_min_ = −0.15 e Å^−3^

### 

Data collection: *APEX2* (Bruker, 2004 ▶); cell refinement: *SAINT* (Bruker, 2004 ▶); data reduction: *SAINT*; program(s) used to solve structure: *SHELXS97* (Sheldrick, 2008 ▶); program(s) used to refine structure: *SHELXL97* (Sheldrick, 2008 ▶); molecular graphics: *PLATON* (Spek, 2009 ▶); software used to prepare material for publication: *SHELXL97* and *PARST* (Nardelli, 1983 ▶).

## Supplementary Material

Crystal structure: contains datablocks I, global. DOI: 10.1107/S1600536809019357/ci2805sup1.cif

Structure factors: contains datablocks I. DOI: 10.1107/S1600536809019357/ci2805Isup2.hkl

Additional supplementary materials:  crystallographic information; 3D view; checkCIF report

## Figures and Tables

**Table 1:** Hydrogen-bond geometry (Å, °) *Cg*1 is the centroid of the C16–C21 ring.

*D*—H⋯*A*	*D*—H	H⋯*A*	*D*⋯*A*	*D*—H⋯*A*
C15—H15*C*⋯*Cg*1^i^	0.96	2.97	3.9108 (26)	167
C23—H23*C*⋯*Cg*1^ii^	0.96	2.86	3.7201 (27)	149

Symmetry codes: (i) 
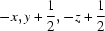
; (ii) 
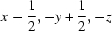
.

**Fig. 1.**
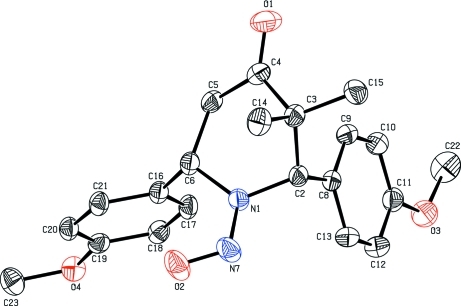
Molecular structure of the title compound. Displacement ellipsoids are drawn at the 30% probability level. H atoms have been omitted for clarity.

**Fig. 2.**
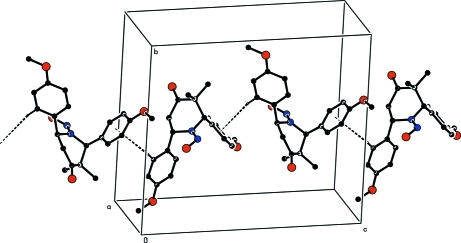
Part of the crystal packing of the title compound, viewed approximately along the a axis. Dashed lines indicate C—H···π interactions. H atoms not involved in the interactions have been omitted.

## supplementary crystallographic information

### Comment

2,6-Disubstituted 4-piperidones are found to have various biological and pharmacological activities (Dimmock *et al.*, 1990; Mutus *et al.*, 1989). Piperidones are also reported to possess analgesic, anti-inflammatory, central nervous system (*CNS*), local anaesthetic, anticancer and antimicrobial activities (Perumal *et al.*, 2001).

The piperidine ring adopts a distorted boat conformation, with puckering parameters (Cremer & Pople, 1975) q2 = 0.613 (2) Å, q3 = -0.123 (2) Å and φ2 = 260.6 (2)° and the asymmetry parameters ΔC_s_(C2) = ΔC_s_(C5) = 21.7 (2)° (Nardelli, 1983). The C8—C13 and C16—C21 phenyl rings are oriented at angles of 88.04 (6)° and 82.38 (7)°, respectively, with the best plane (N1/C3/C4/C6) through the piperidine ring. The C14 methyl group is oriented axially [N1—C2—C3—C14 = 53.8 (2)°] while the C15 methyl group is oriented equatorially [N1—C2—C3—C15 = 173.3 (2)°] to the piperidinone ring. The sum of bond angles around N1 [358.1°] shows that the atom N1 is in *sp*^2 ^ hybridzed state. There is a delocalization between the lone pair of electrons and the hetero π-electrons of the nitroso group.

The packing of the molecules in the crystal is through C—H··· π interactions.

### Experimental

To a solution of r-2,c-6-bis(4-methoxyphenyl)-c-3,t-3-dimethylpiperidin-4-one (1.69 g, 5 mmol) in chloroform (10 ml) was added with conc. HCl (1.5 ml) and water (1.5 ml) and while stirring, solid NaNO_2_ (0.84 g,12 mmol) was added in portions over the period of 0.5 h. The solution was stirred at room temperature for another 0.5 h. The organic layer was washed with water, saturated with aqueous NaHCO_3_ and dried over anhydrous Na_2_SO_4_. The resulting solution was concentrated and the residue was crystallized from ethanol.

### Refinement

H atoms were positioned geometrically (C-H = 0.93–0.98 Å) and allowed to ride on their parent atoms, with *U*_iso_(H) = 1.5*U*_eq_(C) for methyl H and 1.2*U*_eq_(C) for other H atoms. In the absence of significant anomalous scattering effects, Friedel pairs were averaged.

### Crystal data

**Table:**

C_21_H_24_N_2_O_4_	*F*(000) = 784
*M**_r_* = 368.42	*D*_x_ = 1.313 Mg m^−^^3^
Orthorhombic, *P*2_1_2_1_2_1_	Mo *K*α radiation, λ = 0.71073 Å
Hall symbol: P 2ac 2ab	Cell parameters from 3211 reflections
*a* = 7.2540 (3) Å	θ = 2.4–30.5°
*b* = 15.0469 (6) Å	µ = 0.09 mm^−^^1^
*c* = 17.0741 (7) Å	*T* = 293 K
*V* = 1863.64 (13) Å^3^	Block, colourless
*Z* = 4	0.30 × 0.25 × 0.20 mm

### Data collection

**Table:**

Bruker Kappa-APEXII CCD area-detector diffractometer	3211 independent reflections
Radiation source: fine-focus sealed tube	2595 reflections with *I* > 2σ(*I*)
graphite	*R*_int_ = 0.026
ω scans	θ_max_ = 30.5°, θ_min_ = 2.4°
Absorption correction: multi-scan (*SADABS*; Sheldrick, 2001)	*h* = −10→10
*T*_min_ = 0.973, *T*_max_ = 0.982	*k* = −21→21
24656 measured reflections	*l* = −24→24

### Refinement

**Table:**

Refinement on *F*^2^	Primary atom site location: structure-invariant direct methods
Least-squares matrix: full	Secondary atom site location: difference Fourier map
*R*[*F*^2^ > 2σ(*F*^2^)] = 0.039	Hydrogen site location: inferred from neighbouring sites
*wR*(*F*^2^) = 0.109	H-atom parameters constrained
*S* = 1.02	*w* = 1/[σ^2^(*F*_o_^2^) + (0.0532*P*)^2^ + 0.2754*P*] where *P* = (*F*_o_^2^ + 2*F*_c_^2^)/3
3211 reflections	(Δ/σ)_max_ = 0.001
244 parameters	Δρ_max_ = 0.20 e Å^−^^3^
0 restraints	Δρ_min_ = −0.15 e Å^−^^3^

### Special details

**Table:**

Geometry. All e.s.d.'s (except the e.s.d. in the dihedral angle between two l.s. planes) are estimated using the full covariance matrix. The cell e.s.d.'s are taken into account individually in the estimation of e.s.d.'s in distances, angles and torsion angles; correlations between e.s.d.'s in cell parameters are only used when they are defined by crystal symmetry. An approximate (isotropic) treatment of cell e.s.d.'s is used for estimating e.s.d.'s involving l.s. planes.
Refinement. Refinement of *F*^2^ against ALL reflections. The weighted *R*-factor *wR* and goodness of fit *S* are based on *F*^2^, conventional *R*-factors *R* are based on *F*, with *F* set to zero for negative *F*^2^. The threshold expression of *F*^2^ > σ(*F*^2^) is used only for calculating *R*-factors(gt) *etc*. and is not relevant to the choice of reflections for refinement. *R*-factors based on *F*^2^ are statistically about twice as large as those based on *F*, and *R*- factors based on ALL data will be even larger.

### Fractional atomic coordinates and isotropic or equivalent isotropic displacement parameters (Å^2^)

**Table:**

	*x*	*y*	*z*	*U*_iso_*/*U*_eq_	
N1	0.0483 (2)	0.52808 (12)	0.21105 (9)	0.0433 (4)	
C2	0.1305 (2)	0.58054 (13)	0.27489 (10)	0.0417 (4)	
H2	0.0276	0.6015	0.3072	0.050*	
C3	0.2206 (3)	0.66398 (14)	0.23982 (11)	0.0453 (4)	
C4	0.3709 (3)	0.63719 (14)	0.18259 (11)	0.0478 (4)	
C5	0.3450 (3)	0.54846 (14)	0.14164 (11)	0.0464 (4)	
H5A	0.4235	0.5054	0.1675	0.056*	
H5B	0.3898	0.5547	0.0884	0.056*	
C6	0.1494 (3)	0.50917 (13)	0.13767 (10)	0.0419 (4)	
H6	0.0840	0.5392	0.0950	0.050*	
N7	−0.1331 (2)	0.51769 (14)	0.21570 (12)	0.0589 (5)	
C8	0.2461 (3)	0.52025 (13)	0.32719 (10)	0.0413 (4)	
C9	0.4369 (3)	0.51945 (15)	0.33242 (11)	0.0461 (4)	
H9	0.5048	0.5589	0.3020	0.055*	
C10	0.5290 (3)	0.46138 (15)	0.38177 (12)	0.0500 (5)	
H10	0.6571	0.4620	0.3837	0.060*	
C11	0.4317 (3)	0.40259 (13)	0.42807 (12)	0.0484 (4)	
C12	0.2405 (3)	0.40122 (16)	0.42284 (13)	0.0562 (5)	
H12	0.1730	0.3611	0.4528	0.067*	
C13	0.1513 (3)	0.45897 (15)	0.37357 (12)	0.0517 (5)	
H13	0.0233	0.4573	0.3710	0.062*	
C15	0.2886 (4)	0.72672 (16)	0.30428 (13)	0.0581 (5)	
H15A	0.3445	0.7781	0.2809	0.087*	
H15B	0.3777	0.6965	0.3362	0.087*	
H15C	0.1863	0.7449	0.3361	0.087*	
C14	0.0774 (3)	0.71461 (16)	0.18933 (14)	0.0578 (5)	
H14A	0.1337	0.7666	0.1673	0.087*	
H14B	−0.0251	0.7319	0.2215	0.087*	
H14C	0.0347	0.6767	0.1479	0.087*	
C16	0.1535 (2)	0.41093 (12)	0.11881 (10)	0.0393 (4)	
C17	0.2309 (3)	0.34891 (14)	0.16965 (10)	0.0449 (4)	
H17	0.2793	0.3677	0.2173	0.054*	
C18	0.2366 (3)	0.26016 (14)	0.15030 (11)	0.0443 (4)	
H18	0.2889	0.2195	0.1848	0.053*	
C19	0.1647 (2)	0.23100 (12)	0.07945 (10)	0.0402 (4)	
C20	0.0887 (3)	0.29189 (13)	0.02797 (11)	0.0430 (4)	
H20	0.0414	0.2731	−0.0199	0.052*	
C21	0.0838 (3)	0.38045 (14)	0.04814 (10)	0.0431 (4)	
H21	0.0322	0.4210	0.0134	0.052*	
C22	0.7010 (4)	0.34767 (19)	0.49303 (17)	0.0719 (7)	
H22A	0.7349	0.3045	0.5318	0.108*	
H22B	0.7358	0.4059	0.5107	0.108*	
H22C	0.7632	0.3346	0.4448	0.108*	
C23	0.1004 (4)	0.10995 (15)	−0.00595 (14)	0.0605 (6)	
H23A	0.1152	0.0466	−0.0089	0.091*	
H23B	0.1638	0.1374	−0.0490	0.091*	
H23C	−0.0283	0.1245	−0.0085	0.091*	
O1	0.5033 (3)	0.68301 (12)	0.16938 (12)	0.0732 (5)	
O2	−0.2050 (2)	0.48520 (14)	0.15739 (11)	0.0753 (5)	
O3	0.5071 (3)	0.34479 (11)	0.48087 (10)	0.0640 (4)	
O4	0.1751 (2)	0.14144 (9)	0.06582 (8)	0.0509 (3)	

### Atomic displacement parameters (Å^2^)

**Table:**

	*U*^11^	*U*^22^	*U*^33^	*U*^12^	*U*^13^	*U*^23^
N1	0.0311 (6)	0.0576 (9)	0.0412 (7)	0.0002 (7)	0.0008 (6)	−0.0093 (7)
C2	0.0358 (8)	0.0543 (10)	0.0348 (8)	−0.0009 (8)	0.0034 (7)	−0.0084 (7)
C3	0.0446 (9)	0.0519 (10)	0.0393 (8)	0.0004 (8)	0.0037 (8)	−0.0051 (8)
C4	0.0461 (10)	0.0531 (11)	0.0442 (9)	−0.0003 (9)	0.0077 (8)	0.0035 (8)
C5	0.0432 (9)	0.0573 (11)	0.0387 (8)	0.0012 (9)	0.0088 (8)	−0.0034 (8)
C6	0.0403 (8)	0.0522 (10)	0.0331 (8)	0.0041 (8)	−0.0015 (7)	−0.0029 (7)
N7	0.0347 (8)	0.0767 (12)	0.0654 (11)	−0.0027 (8)	−0.0038 (8)	−0.0179 (10)
C8	0.0390 (8)	0.0525 (11)	0.0323 (7)	−0.0055 (8)	0.0015 (7)	−0.0049 (8)
C9	0.0401 (9)	0.0592 (11)	0.0389 (9)	−0.0079 (9)	0.0024 (7)	0.0025 (9)
C10	0.0432 (9)	0.0621 (12)	0.0449 (10)	−0.0046 (9)	−0.0008 (9)	−0.0002 (9)
C11	0.0600 (12)	0.0453 (10)	0.0400 (9)	−0.0028 (9)	0.0003 (9)	−0.0036 (8)
C12	0.0600 (12)	0.0569 (12)	0.0516 (11)	−0.0158 (10)	0.0075 (10)	0.0059 (10)
C13	0.0410 (9)	0.0638 (13)	0.0504 (10)	−0.0121 (10)	0.0056 (9)	0.0009 (10)
C15	0.0579 (12)	0.0581 (12)	0.0582 (12)	−0.0077 (10)	0.0029 (11)	−0.0143 (10)
C14	0.0607 (13)	0.0600 (12)	0.0527 (11)	0.0116 (11)	−0.0001 (11)	−0.0026 (10)
C16	0.0356 (8)	0.0494 (9)	0.0329 (7)	0.0046 (8)	−0.0010 (7)	−0.0024 (7)
C17	0.0425 (9)	0.0604 (11)	0.0319 (8)	0.0043 (9)	−0.0063 (7)	−0.0014 (8)
C18	0.0390 (9)	0.0555 (11)	0.0383 (8)	0.0070 (8)	−0.0033 (7)	0.0074 (8)
C19	0.0324 (8)	0.0480 (9)	0.0403 (8)	0.0009 (7)	0.0050 (7)	0.0008 (7)
C20	0.0420 (9)	0.0533 (10)	0.0336 (8)	0.0022 (8)	−0.0047 (7)	−0.0021 (8)
C21	0.0432 (9)	0.0523 (10)	0.0337 (8)	0.0060 (8)	−0.0065 (7)	0.0020 (8)
C22	0.0729 (16)	0.0707 (16)	0.0723 (15)	0.0205 (14)	−0.0010 (14)	0.0133 (13)
C23	0.0649 (13)	0.0527 (12)	0.0638 (13)	−0.0002 (11)	−0.0052 (12)	−0.0134 (11)
O1	0.0684 (10)	0.0652 (10)	0.0858 (13)	−0.0185 (9)	0.0299 (10)	−0.0047 (9)
O2	0.0420 (8)	0.1026 (14)	0.0812 (11)	0.0025 (9)	−0.0132 (8)	−0.0327 (11)
O3	0.0740 (11)	0.0592 (9)	0.0589 (9)	−0.0050 (9)	−0.0057 (9)	0.0118 (8)
O4	0.0543 (8)	0.0467 (7)	0.0516 (7)	0.0036 (6)	−0.0011 (7)	0.0001 (6)

### Geometric parameters (Å, °)

**Table:**

N1—N7	1.328 (2)	C13—H13	0.93
N1—C2	1.472 (2)	C15—H15A	0.96
N1—C6	1.479 (2)	C15—H15B	0.96
C2—C8	1.524 (3)	C15—H15C	0.96
C2—C3	1.537 (3)	C14—H14A	0.96
C2—H2	0.98	C14—H14B	0.96
C3—C4	1.519 (3)	C14—H14C	0.96
C3—C15	1.532 (3)	C16—C21	1.386 (2)
C3—C14	1.550 (3)	C16—C17	1.393 (3)
C4—O1	1.204 (2)	C17—C18	1.376 (3)
C4—C5	1.519 (3)	C17—H17	0.93
C5—C6	1.539 (3)	C18—C19	1.389 (3)
C5—H5A	0.97	C18—H18	0.93
C5—H5B	0.97	C19—O4	1.370 (2)
C6—C16	1.513 (3)	C19—C20	1.384 (3)
C6—H6	0.98	C20—C21	1.377 (3)
N7—O2	1.225 (2)	C20—H20	0.93
C8—C9	1.387 (3)	C21—H21	0.93
C8—C13	1.396 (3)	C22—O3	1.423 (3)
C9—C10	1.385 (3)	C22—H22A	0.96
C9—H9	0.93	C22—H22B	0.96
C10—C11	1.380 (3)	C22—H22C	0.96
C10—H10	0.93	C23—O4	1.421 (3)
C11—O3	1.367 (3)	C23—H23A	0.96
C11—C12	1.390 (3)	C23—H23B	0.96
C12—C13	1.372 (3)	C23—H23C	0.96
C12—H12	0.93		
			
N7—N1—C2	114.87 (16)	C12—C13—H13	118.9
N7—N1—C6	121.28 (17)	C8—C13—H13	118.9
C2—N1—C6	121.97 (14)	C3—C15—H15A	109.5
N1—C2—C8	109.72 (15)	C3—C15—H15B	109.5
N1—C2—C3	108.78 (15)	H15A—C15—H15B	109.5
C8—C2—C3	118.74 (16)	C3—C15—H15C	109.5
N1—C2—H2	106.3	H15A—C15—H15C	109.5
C8—C2—H2	106.3	H15B—C15—H15C	109.5
C3—C2—H2	106.3	C3—C14—H14A	109.5
C4—C3—C15	113.24 (18)	C3—C14—H14B	109.5
C4—C3—C2	109.81 (16)	H14A—C14—H14B	109.5
C15—C3—C2	111.14 (16)	C3—C14—H14C	109.5
C4—C3—C14	104.71 (16)	H14A—C14—H14C	109.5
C15—C3—C14	108.22 (18)	H14B—C14—H14C	109.5
C2—C3—C14	109.47 (17)	C21—C16—C17	117.90 (17)
O1—C4—C5	121.09 (19)	C21—C16—C6	120.07 (16)
O1—C4—C3	122.78 (19)	C17—C16—C6	122.00 (16)
C5—C4—C3	116.13 (17)	C18—C17—C16	120.84 (17)
C4—C5—C6	118.19 (17)	C18—C17—H17	119.6
C4—C5—H5A	107.8	C16—C17—H17	119.6
C6—C5—H5A	107.8	C17—C18—C19	120.29 (18)
C4—C5—H5B	107.8	C17—C18—H18	119.9
C6—C5—H5B	107.8	C19—C18—H18	119.9
H5A—C5—H5B	107.1	O4—C19—C20	124.43 (17)
N1—C6—C16	112.22 (16)	O4—C19—C18	115.98 (17)
N1—C6—C5	110.23 (15)	C20—C19—C18	119.58 (18)
C16—C6—C5	111.48 (16)	C21—C20—C19	119.48 (17)
N1—C6—H6	107.6	C21—C20—H20	120.3
C16—C6—H6	107.6	C19—C20—H20	120.3
C5—C6—H6	107.6	C20—C21—C16	121.90 (17)
O2—N7—N1	114.82 (19)	C20—C21—H21	119.1
C9—C8—C13	116.69 (19)	C16—C21—H21	119.1
C9—C8—C2	126.27 (18)	O3—C22—H22A	109.5
C13—C8—C2	117.03 (17)	O3—C22—H22B	109.5
C10—C9—C8	121.70 (19)	H22A—C22—H22B	109.5
C10—C9—H9	119.2	O3—C22—H22C	109.5
C8—C9—H9	119.2	H22A—C22—H22C	109.5
C11—C10—C9	120.41 (19)	H22B—C22—H22C	109.5
C11—C10—H10	119.8	O4—C23—H23A	109.5
C9—C10—H10	119.8	O4—C23—H23B	109.5
O3—C11—C10	125.5 (2)	H23A—C23—H23B	109.5
O3—C11—C12	115.6 (2)	O4—C23—H23C	109.5
C10—C11—C12	118.9 (2)	H23A—C23—H23C	109.5
C13—C12—C11	120.0 (2)	H23B—C23—H23C	109.5
C13—C12—H12	120.0	C11—O3—C22	118.20 (19)
C11—C12—H12	120.0	C19—O4—C23	116.96 (16)
C12—C13—C8	122.26 (19)		
			
N7—N1—C2—C8	110.4 (2)	C3—C2—C8—C13	164.11 (17)
C6—N1—C2—C8	−85.0 (2)	C13—C8—C9—C10	−0.6 (3)
N7—N1—C2—C3	−118.2 (2)	C2—C8—C9—C10	−179.66 (17)
C6—N1—C2—C3	46.4 (2)	C8—C9—C10—C11	−0.6 (3)
N1—C2—C3—C4	−60.6 (2)	C9—C10—C11—O3	−177.43 (19)
C8—C2—C3—C4	65.7 (2)	C9—C10—C11—C12	1.6 (3)
N1—C2—C3—C15	173.29 (16)	O3—C11—C12—C13	177.69 (18)
C8—C2—C3—C15	−60.3 (2)	C10—C11—C12—C13	−1.4 (3)
N1—C2—C3—C14	53.80 (19)	C11—C12—C13—C8	0.2 (3)
C8—C2—C3—C14	−179.83 (16)	C9—C8—C13—C12	0.8 (3)
C15—C3—C4—O1	−26.9 (3)	C2—C8—C13—C12	179.92 (19)
C2—C3—C4—O1	−151.7 (2)	N1—C6—C16—C21	122.51 (18)
C14—C3—C4—O1	90.8 (3)	C5—C6—C16—C21	−113.29 (19)
C15—C3—C4—C5	153.57 (19)	N1—C6—C16—C17	−59.4 (2)
C2—C3—C4—C5	28.7 (2)	C5—C6—C16—C17	64.8 (2)
C14—C3—C4—C5	−88.7 (2)	C21—C16—C17—C18	−0.4 (3)
O1—C4—C5—C6	−158.8 (2)	C6—C16—C17—C18	−178.54 (18)
C3—C4—C5—C6	20.8 (3)	C16—C17—C18—C19	−0.1 (3)
N7—N1—C6—C16	−69.4 (2)	C17—C18—C19—O4	−179.38 (18)
C2—N1—C6—C16	127.05 (19)	C17—C18—C19—C20	0.6 (3)
N7—N1—C6—C5	165.7 (2)	O4—C19—C20—C21	179.33 (18)
C2—N1—C6—C5	2.1 (2)	C18—C19—C20—C21	−0.7 (3)
C4—C5—C6—N1	−37.0 (2)	C19—C20—C21—C16	0.2 (3)
C4—C5—C6—C16	−162.27 (16)	C17—C16—C21—C20	0.3 (3)
C2—N1—N7—O2	170.12 (19)	C6—C16—C21—C20	178.52 (18)
C6—N1—N7—O2	5.4 (3)	C10—C11—O3—C22	3.6 (3)
N1—C2—C8—C9	109.0 (2)	C12—C11—O3—C22	−175.5 (2)
C3—C2—C8—C9	−16.9 (3)	C20—C19—O4—C23	−0.7 (3)
N1—C2—C8—C13	−70.0 (2)	C18—C19—O4—C23	179.28 (18)

### Hydrogen-bond geometry (Å, °)

**Table:**

*D*—H···*A*	*D*—H	H···*A*	*D*···*A*	*D*—H···*A*
C15—H15C···Cg1^i^	0.96	2.97	3.9108 (26)	167
C23—H23C···Cg1^ii^	0.96	2.86	3.7201 (27)	149

Symmetry codes: (i) −*x*, *y*+1/2, −*z*+1/2; (ii) *x*−1/2, −*y*+1/2, −*z*.
